# Meteorological disaster disturbances on the main crops in the north‒south transitional zone of China

**DOI:** 10.1038/s41598-024-59106-3

**Published:** 2024-04-17

**Authors:** Yanan Li, Xi Wang, Guangrui Xing, Dongfeng Wang

**Affiliations:** 1 Key Laboratory of Geospatial Technology for the Middle and Lower Yellow River Regions (Henan University), Ministry of Education & College of Geography and Environmental Science, Henan University, Kaifeng, 475004 China; 2 Zhengzhou Tourism College, Zhengzhou, 451464 China; 3 Key Research Institute of Yellow River Civilization and Sustainable Development & Collaborative Innovation Center on Yellow River Civilization Jointly Built by Henan Province and Ministry of Education, Henan University, Kaifeng, 475001 China; 4 School of Culture and Tourism, Henan University, Kaifeng, 475001 China

**Keywords:** Winter wheat, Summer maize, Extreme precipitation, Drought, North–South transitional zone, Disaster disturbance, Climate change, Natural hazards

## Abstract

Global climate change, with warming as its main feature, has altered the spatial-temporal evolution of factors such as precipitation and temperature that can cause meteorological disasters. The complex and changeable climate has led to frequent natural disasters, while the frequency and intensity of extreme climate events have also significantly increased, posing an enormous threat to societal production and human life. As the most important geoecological transitional zone of mainland China, the stability of agricultural production in China’s north–south transitional zone is crucial for ensuring food security under climate change. With the use of daily precipitation and potential evapotranspiration data from 1961 to 2018, this study focused on analysing disturbances such as extreme precipitation and drought disasters at different time scales during the winter wheat and summer maize growing seasons in the north–south transitional zone of China from an agricultural production perspective and attempted to answer the following questions: first, from an agricultural production perspective, what are the temporal and spatial distribution patterns of extreme precipitation and arid climate events in the north–south transitional zone? Second, which areas are at high risk of being disturbed by different types of meteorological disasters and require increased attention? The results indicated that (1) in terms of the overall temporal variation, the degree of extreme precipitation and drought stress faced by agricultural production in the region is decreasing. However, the temporal variation at each station in the north–south transitional zone was not completely consistent with the overall trend, and both increasing and decreasing trends were observed. The sites exhibiting an increase overlapped with typical regions of the north–south transitional zone to varying degrees, indicating that the typical regions represented not only theoretical potential risk areas under climate change but also suffered from meteorological disaster disturbances. (2) The precipitation distribution during the winter wheat growth period in the south–north transitional zone was uneven and varied significantly. High values of extreme precipitation indices during the winter wheat growth period were mainly concentrated in the southern part of the eastern section of the north‒south transitional zone. The precipitation distribution during the summer maize growth period significantly differed, with the highest amount of heavy rain and largest number of rainstorm days concentrated in the southeastern part of the north‒south transitional zone. The spatial distribution of the drought frequency in the north–south transitional zone, as indicated by the monthly standardized precipitation evapotranspiration index (SPEI_1_), showed that the areas with high total drought frequencies were mainly concentrated in northeast Jiangsu, southeast Henan, and north Anhui, which primarily experienced light drought. The central part of Jiangsu Province exhibited a high frequency of moderate drought, while southern Jiangsu Province and southwestern Shaanxi Province were prone to severe drought. Additionally, southeastern Hebei and eastern Henan were identified as areas with a high frequency of extreme drought. Finally, the central region of Sichuan Province was characterized by both severe and extreme drought conditions. Based on the SPEI_12_-derived spatial distribution of the drought frequency in the north–south transitional zone, the areas with a high total drought frequency were mainly concentrated in central and eastern Henan, southeast Shaanxi, southeast Shandong, and central Sichuan, which primarily experienced light to moderate drought. The northwestern part of Jiangsu, the southern part of Hebei, and the western part of Shandong are regions with a high frequency of severe drought, while the eastern part of Henan is an area with high frequencies of both severe and extreme drought. (3) High-value areas of extreme precipitation and drought disturbance in the north–south transitional zone overlapped with the edge of the transitional zone to varying degrees. Approximately 63.58% of the north‒south transitional zone of China was characterized by moderate or high stress levels, primarily concentrated along the southern boundary and central core area, and nearly 39.5% of all counties experienced two or more types of disaster stresses.

## Introduction

The findings of previous assessment reports from the Intergovernmental Panel on Climate Change (IPCC) have confirmed the indisputable nature of global warming^1–3^. The first working group of the sixth evaluation report of the IPCC (IPCC AR6 WGI) released the 2021 Climate Change–Natural Science Foundations, which noted that human activities have caused warming of the atmosphere, oceans, and land. It is expected that climate system warming will continue through the middle of this century. Global climate change, which is characterized by warming, has altered the spatial-temporal evolution of factors that can cause meteorological disasters, such as precipitation and temperature^4^. The complex and ever-changing climate has led to frequent natural disasters, and the frequency and intensity of extreme climate events have significantly increased, posing a considerable threat to the productivity, life, and safety of human society^5,6^. Agriculture is the most directly vulnerable sector affected by climate change, and food shortages caused by climate change may occur faster and earlier than the effects of rising sea levels^7^. Identifying vulnerable agricultural production areas under climate change and identifying the key factors influencing regional agricultural production vulnerability are crucial for informing agricultural adaptation behaviour and enhancing adaptability to climate change. This is very important for ensuring food security under climate change, maintaining agricultural production stability, promoting farmers’ income growth, and improving farmers’ living standards.

The north‒south transitional zone of China is the most important geographical ecotone of the Chinese mainland. Because of its high environmental complexity, biodiversity, climate sensitivity and transition, it is important to study the geographical pattern of China, the evolution of biota, and the response of geographical elements to climate change, which represents one of the key areas for breakthroughs in climate change research^8^. In 1908, Zhang Xiangwen first defined the Qinling–Huaihe line as the natural geographical boundary between north and south^9^. Over the subsequent century, geographic researchers have continuously refined indicators for delineating this boundary and explored its precise location^8–13^. Further research has revealed a crucial fact: the north‒south boundary is not a rigid line but instead encompasses a transitional zone with varying widths over time. The range and boundaries of this transitional zone will shift under climate change. Consequently, several new questions arise concerning the north–south transitional zone: How can its position be determined? How can the direction, extent and boundaries of this transitional zone be determined?

Using raster calculations in ArcGIS and the mean–standard deviation method in statistics, we determined the boundaries and extent of the north–south transitional zone in China. This was achieved through a transitional zone of varying widths. From 1951 to 2018, the isolines of each determined climate index exhibited fluctuations of different amplitudes with alternating cold and warm periods, with the cold period advancing southwards and the warm period advancing northwards^14^. The vulnerability of agricultural production in this region will be exacerbated by climate instability. Notably, regions within marginal transitional zones should be focused on as representative areas affected by climate change.

Winter wheat and summer maize are the main food crops in the north‒south transitional zone. Extreme precipitation events during the growing season constitute the most direct negative factor affecting winter wheat and summer maize production^15^. These events can cause flooding and moisture damage during the growing season, resulting in reduced yields and even root system death. If these events occur during the mature period, they can also affect crop quality, resulting in high yields but not high harvests^16^. Drought is also a major agricultural meteorological disaster that affects winter wheat and summer maize. Drought during the entire growing season or at critical growth stages can affect crop growth, hindering the grain filling process and significantly reducing yields^17–20^.

To explore the impacts of meteorological disturbances on agricultural production in the north‒south transitional zone of China, extreme precipitation and drought were selected as the factors causing disasters in agricultural production^21–23^. This study focused on analysing the temporal variation and spatial distributions of extreme precipitation and arid climate events in the north‒south transitional zone by attempting to answer the following questions: first, what are the temporal and spatial distribution patterns of extreme precipitation and arid climate events in this transitional zone from an agricultural production perspective? Second, which areas are at high risk of being disturbed by different types of meteorological disasters and require increased attention?

## Materials and methods

### Study area

**Figure 1:**
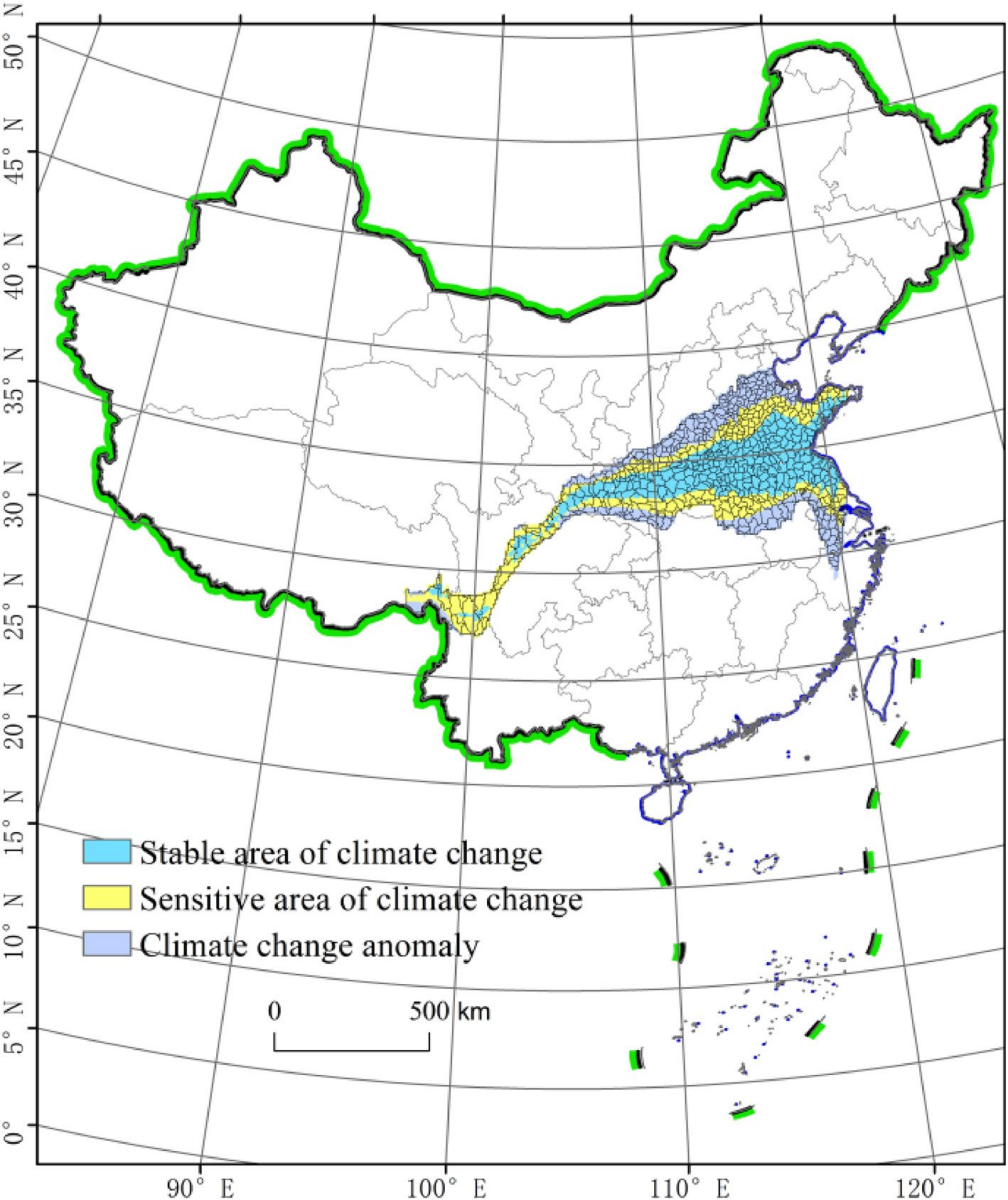
The north–south transitional zone of China. Study area, the north–south transitional zone of China, highlighting the stable area of climate change in blue, sensitive area of climate in yellow, climate change anomaly in purple, the base map used in the maps in figure number 1, 2, 6, 7, 8, 9, 10, 11, 12, 13, 14 are drawn from the Standard map service system of the Ministry of Natural Resources of China (Drawing review No. GS (2019) 1697, http://bzdt.ch.mnr.gov.cn/download.html), and the base map has not been modified. Composed using ESRI ArcGIS 10.2 Software. *This work is licensed under a Creative Commons by Attribution (CC BY 4.0) license. **ESRI, China.

The north‒south transitional zone of China is the most important geographical ecotone of mainland China. This zone is not only a complete geographical and geomorphic unit but also the most important geographical and agricultural boundary in China that separates warm temperate and subtropical zones. From a spatial perspective, the traditional north‒south transitional zone (with the Qinling Mountains and Huaihe River) is where the boundary crosses Shaanxi, Gansu, Henan, Hubei, Sichuan, and other provinces. It approximately starts in Wudu in southern Gansu in the west and extends along the ridge of the Qinling Mountains to the Funiu Mountains in Henan. This description provides an overall and relatively general scope. In contrast to the traditional north–south transitional zone of China, the north–south transitional zone in this study exhibited a clearly defined boundary and scope. It was based on the purpose of serving agricultural production. Historical climate observation data, combined with the selection of climate elements as indicators of the boundaries of the north‒south transitional zone, were used to divide this transitional zone into three parts^14^. The northernmost boundary of China′s transitional zone passed through Lixian, Yaoxian, Hancheng, Anze, Shexian and Jinghai Counties from west to east. The southernmost boundary of China’s transitional zone passed through Beichuan, Ningqiang, Xixiang, Fangxian, Xichuan, Luoshan, Shangcheng, Dingyuan and Lin’an Counties from west to east. Among the 637 counties extracted in this area, 256 counties were located in the climate change stability area of the north‒south transitional zone, while 187 counties were located in the climate change-sensitive area (Fig. 1).

### Materials and statistical methods

#### Crop growing season statistics

**Figure 2:**
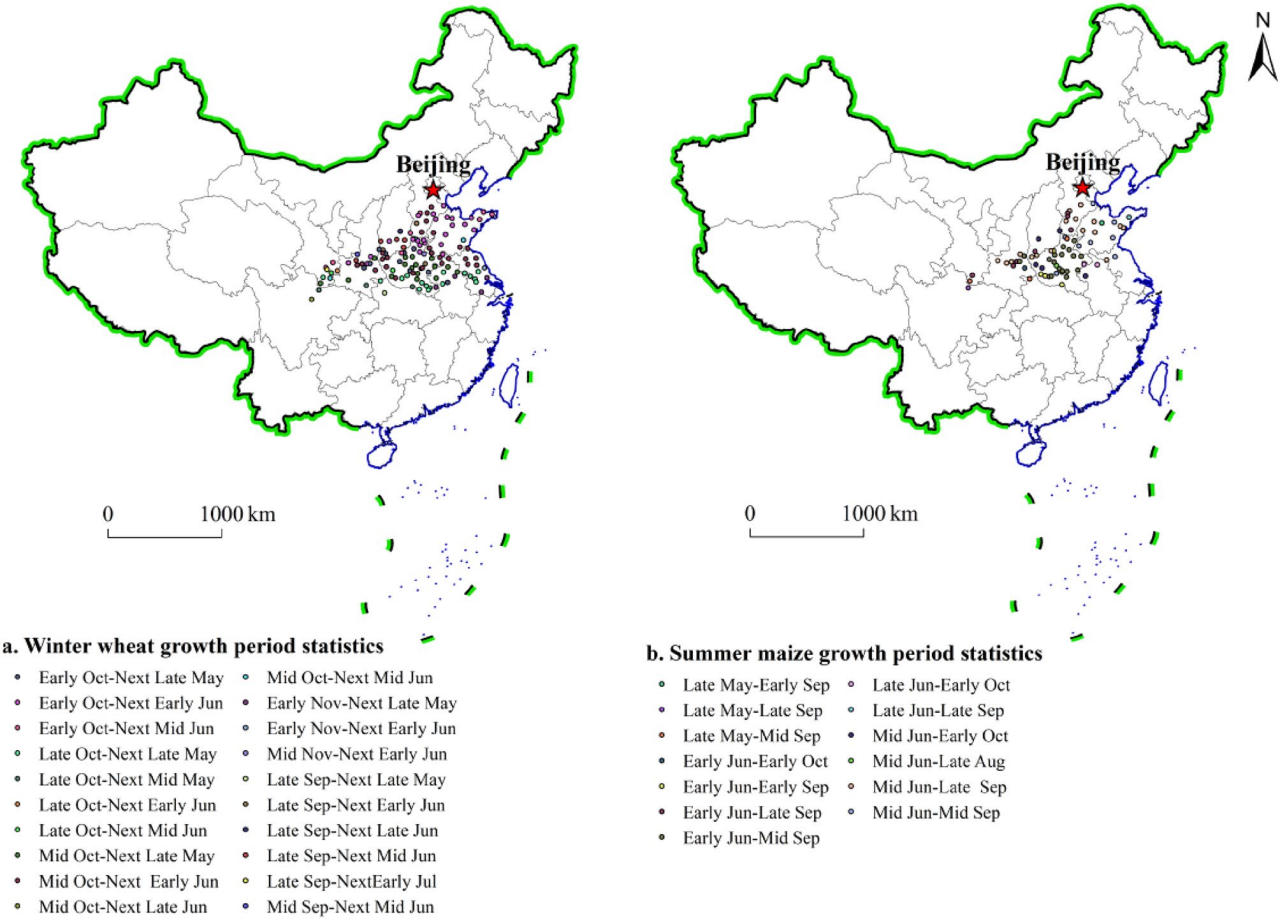
Statistics for the winter wheat and summer maize growing seasons in the north–south transitional zone of China.

The China Meteorological Science Data Sharing Service Network was used in this study (http://data.cma.cn/site/index.html). Crop growth and development data from 778 agricultural meteorological stations in China for the 1991 to 2014 period included statistics on the growing seasons of winter wheat and summer maize in various regions. The average sowing and ripening dates of winter wheat and summer maize at each station were calculated based on the sowing and ripening dates, respectively, of winter wheat and summer maize over time. After observing the developmental stages of winter wheat and summer maize in various regions, it was found that due to climate change, the sowing and ripening dates of winter wheat in various regions of China have advanced or been delayed over the years. With an 80% guarantee rate, there was an approximately 5-day advance or delay. To include disastrous weather events, the time interval encompassing the sowing period and 5 days before and after the maturity period was defined as the growing season for winter wheat and summer maize in this study^24^. The statistical periods for the winter wheat and summer maize growing seasons in the north‒south transitional zone of China included 20 and 13 stages, respectively (Fig. 2).

#### Statistics of agricultural meteorological disaster data

Daily precipitation and evapotranspiration data from the China Meteorological National Meteorological Stations were provided by the Resource and Environment Science Data Center of the Chinese Academy of Sciences (http://www.resdc.cn/data.aspx). The number of national meteorological stations increased from 182 in 1951 to 2421 in 2018. Upon data analysis, we identified varying instances of missing observations for different meteorological variables across the different years. To ensure data continuity and completeness, we excluded and interpolated missing data based on climate factors during calculations. For instance, when calculating the annual precipitation, we removed meteorological stations with continuous missing data exceeding seven days within a year and interpolated those with cumulative intermittent missing periods not exceeding 30 days.

1. Extreme precipitation events

The fifth report of the IPCC noted that the frequency and intensity of extreme climate events worldwide have increased due to the impacts of climate change. Extreme precipitation is a typical manifestation of extreme weather, causing frequent meteorological disasters and increased economic and societal losses. This phenomenon has become one of the most complex challenges facing humanity^25,26^. In this study, the general state of precipitation during the growing seasons of winter wheat and summer maize was represented by the number of rainy days, and the extreme state of precipitation was represented by the number of rainy days, the number of rainstorm days, the frequency of continuous rain, the maximum process rainfall, and the largest number of consecutive rainy days (Table 1). The above indicators collectively represent the level of stress caused by extreme precipitation events during the growing seasons of winter wheat and summer maize in typical areas of the north‒south transitional zone. The definitions of the extreme precipitation indicators are listed in Table 1^27–29^.

(2) Drought

**Table 1:** Definition of the extreme precipitation index.

Extreme precipitation index	Indicator definition
Precipitation days	Daily precipitation ≥ 1 mm Days/d
Heavy rain days	Daily precipitation ≥ 25 mm Days/d
Rainstorm days	Daily precipitation ≥ 50 mm Days/d
Continuous rain frequency	Days with continuous precipitation for 3 days or more and process rainfall ≥ 40 mm/d
Maximum continuous rainy process rainfall	Continuous precipitation for 3 days or more and process rainfall ≥ 40 mm Maximum precipitation/mm
Maximum continuous rainy days	Maximum number of days with continuous precipitation for 3 days or more and process rainfall ≥ 40 mm/d

The Classification Standard of Meteorological Drought formulated by the China Meteorological Administration divides the SPEI into five grades: no drought, slight drought, moderate drought, severe drought, and extreme drought (Table 2). The SPEI encompasses multiple time scales. On a one-month time scale, the drought index can reflect subtle changes in droughts and floods, while the 12-month time scale can reflect the overall annual drought situation. Therefore, in this study, the SPEI was calculated at two scales, namely, one- and twelve-month scales, denoted as SPEI_1_ and SPEI_12,_ respectively.

**Table 2:** Classification standards for meteorological drought levels.

Drought level	Range values of SPEI
No drought	SPEI ≥ − 0.5
Mild drought	− 1.0 < SPEI ≤ − 0.5
Moderate drought	− 1.5 < SPEI ≤ − 1.0
Severe drought	− 2.0 < SPEI ≤ − 1.5
Extreme drought	SPEI ≤ − 2.0

According to the Classification Standard for Meteorological Drought Levels, the SPEI_1_ and SPEI_12_ in China from 1961 to 2018 were calculated, and drought data for 696 months and 58 years were collected from various meteorological stations. The frequency and total frequency of light drought, moderate drought, severe drought, and extreme drought at these two time scales during the study period were calculated to characterize the drought intensity in the study area (Table 3).

**Table 3:** Definition of drought meteorological disaster indicators.

Drought indicators	Indicator definition
Drought intensity	The number of occurrences of a certain level of drought during the research period

### Climate inclination rate

The climate inclination rate refers to the multiyear change trend of climate elements. When calculating the change trend, the least squares method can be used to calculate the linear regression coefficient *b* of sample* x*_*i*_ at time *t*, and the change in each element can be expressed by the following linear equation:

1 $$ x_{i} = {\text{a}} + {\text{b}}t_{i} \quad i = 1, 2, 3, \ldots ,{\text{n}} $$

where *x* is the factor value, *a* is the constant term, *b* is the regression coefficient, *i* is the year of the time series, and the climate inclination rate is equal to ten times the regression coefficient b. For b > 0, *x* increases with increasing time *t*. In contrast, for b < 0, *x* shows a decreasing trend.

### Statistical methods

#### Standardized evapotranspiration index (SPEI)

- ① The potential evapotranspiration was calculated using the FAO Penman‒Monteith model^30^.
- ② The difference between the monthly precipitation and potential evapotranspiration was calculated as the water deficit:
  2 $${D}_{j}={P}_{j}-PE{T}_{j}$$
  where ${{\text{D}}}_{{\text{j}}}$ is the water deficit in month $j$, ${{\text{P}}}_{{\text{j}}}$ is the precipitation in month $j$, and ${{\text{PET}}}_{{\text{j}}}$ is the potential evapotranspiration in month $j$.
- ③ Based on the linear decreasing weight scheme^31^, a series of accumulated water and deficits was established at different time scales:
  3 $$ \left\{ {\begin{array}{*{20}l} {X_{i,j}^{k} = \sum\nolimits_{l = 13 - k + j}^{12} {D_{i - 1,l} } + \sum\nolimits_{l = 1}^{j} {D_{i,l} } } \hfill & {j < k} \hfill \\ {X_{i,j}^{k} = \sum\nolimits_{l = j - k}^{j} {D_{i,l} } } \hfill & {j \ge k} \hfill \\ \end{array} } \right. $$
  where ${X}_{i,j}^{k}$ is the accumulated water deficit in month $l$ of year $i$ at the monthly scale and ${D}_{i,l}$ is the water deficit in month $l$ of year $i$.
- ④ Due to the negative value of the original cumulative water deficit, it was necessary to introduce a three-parameter log-logistic probability distribution function to calculate the cumulative water deficit probability distribution^32^.
  4 $$F(X)={\left[1+{\left(\frac{\alpha }{X-\gamma }\right)}^{\beta }\right]}^{-1}$$
  where $\alpha$, $\beta$ and $\gamma$ denote the scale, shape and position, respectively, and can be expressed as follows:
  5 $$\beta =\frac{2{w}_{1}-{w}_{0}}{\left(6{w}_{1}-{w}_{0}-6{w}_{2}\right)}$$
  6 $$\alpha =\frac{\left({w}_{0}-2{w}_{1}\right)\beta }{\Gamma (1+1/\beta )\Gamma (1-1/\beta )}$$
  7 $$\gamma ={w}_{0}-\alpha\Gamma (1+1/\beta )\Gamma (1-\frac{1}{\beta })$$
  8 $${w}_{s}=\frac{1}{n}\sum_{q=1}^{n} {\left(1-\frac{q-0.35}{n}\right)}^{s}{X}_{q}$$
  where ${w}_{s}$ is the probability weight moment, $s$= 0,1,2, and $q$ is the ordinal number of the accumulated water deficits in ascending order, which satisfies ${X}_{1}\le {X}_{2}\le \cdots \le {X}_{n}$. Moreover, it i $\Gamma (\beta )$ s the Gamma function.
- ⑤ The probability distribution of the cumulative water deficit series for each month of standardized treatment is ${\text{F}}({\text{X}})$, where ${\text{p}}$ is the probability of ${{\text{X}}}_{{\text{I}},{\text{j}}}^{{\text{k}}}$:
  9 $$ p = 1 - F(X) $$
  IF $$p \le 0.5,w = \sqrt { - 2\ln p}$$, then
  10 $$SPEI=w-\frac{{C}_{0}+{C}_{1}w+{C}_{2}{w}^{2}}{1+{d}_{1}w+{d}_{2}{w}^{2}+{d}_{3}{w}^{3}}$$
  IF $$p>0.5,w=\sqrt{-2{\text{ln}}(1-p)}$$, then
  11 $$SPEI=\frac{{C}_{0}+{C}_{1}w+{C}_{2}{w}^{2}}{1+{d}_{1}w+{d}_{2}{w}^{2}+{d}_{3}{w}^{3}}-w$$
  where $${{\text{C}}}_{0}$$= 2.515517 $$, {{\text{C}}}_{1}$$=0.802853 $$, {{\text{C}}}_{2}$$= 0.010328 $$, {{\text{d}}}_{1}$$= 1.432788 $$, {{\text{d}}}_{3}$$= 0.189269, and $${{\text{d}}}_{3}$$= 0.001308.

## Results

### Temporal variability in major meteorological disasters in the north–south transitional zone

#### Extreme precipitation changes during the winter wheat growing season

**Figure 3:**
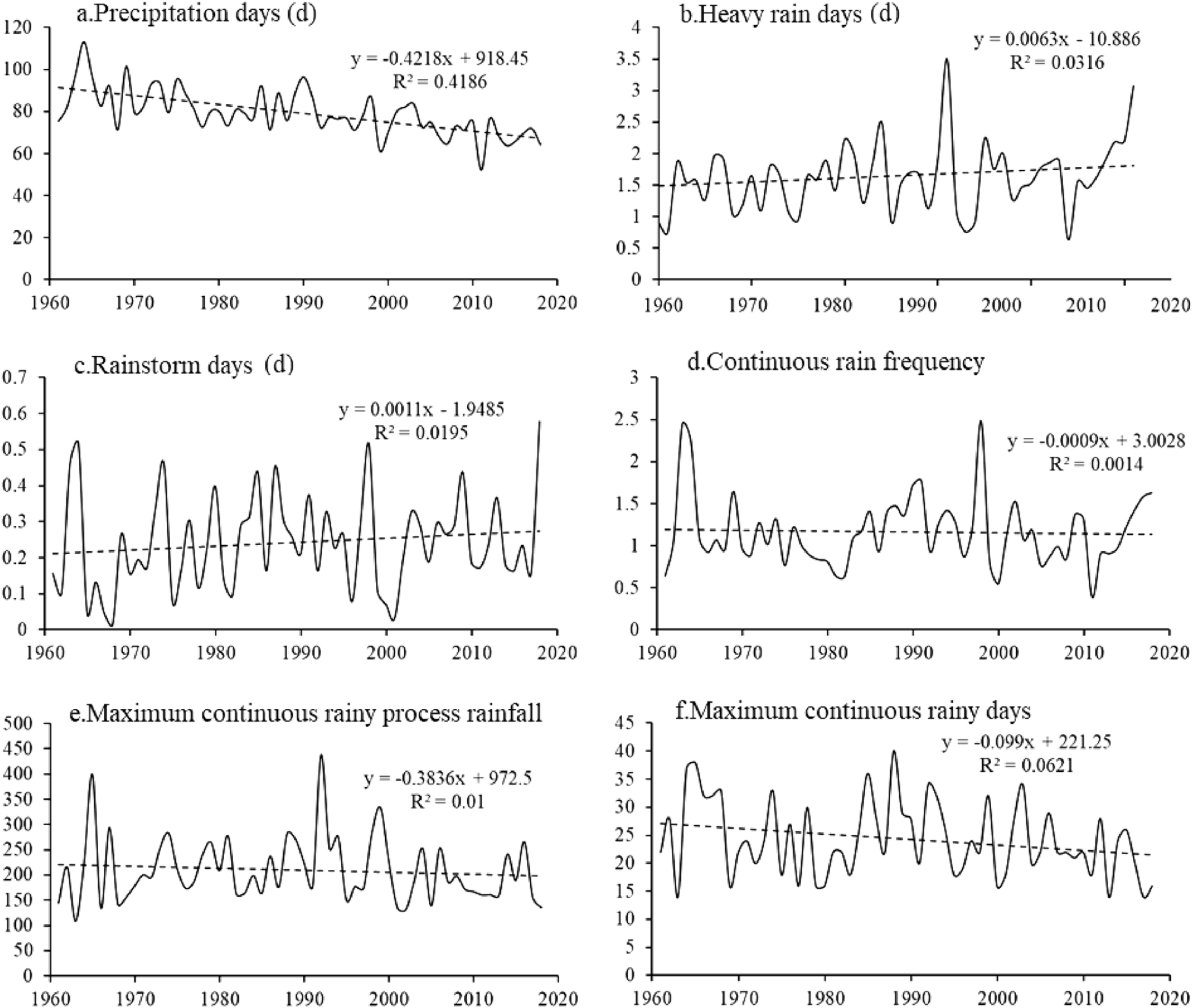
Extreme precipitation trends during the winter wheat growing season from 1961 to 2018.

Figure 3 shows the interannual trend of the extreme precipitation index during the winter wheat growing season in the north‒south transitional zone from 1961 to 2018. Over the past 58 years, the number of heavy rain days, the number of rainstorm days and the frequency of continuous rain slightly varied, while the number of precipitation days, the maximum continuous rainfall and the largest number of consecutive rainfall days exhibited high variability. The number of heavy rain days and rainstorm days increased. The year with the greatest number of heavy rain days was 1998, with 3.5 d, while the year with the smallest number was 2011, with 0.64 d. The year with the greatest number of rainstorm days was 2018, with 0.58 d, while the year with the smallest number was 1968, with 0.01 d.

The frequency of continuous rain decreased. The year with the most continuous rain was 1998 (2.48), while the year with the least continuous rain was 2011 (0.39). Moreover, the number of precipitation days decreased. The year with the most precipitation days was 1964, in which the total precipitation reached 112.98 mm, while the year with the smallest number of precipitation days was 2011, in which the precipitation reached only 52.26 mm. Although the maximum number of rainfall events and the greatest number of consecutive continuous rainy days decreased, the annual variability greatly differed. The single maximum rainfall event on continuous rainy days occurred in 1992, reaching 438 mm, which was four times greater than the maximum rainfall event in 1963. The year with the greatest number of consecutive rainy days was 1988, at 40 d, and the smallest number of consecutive rainy days occurred in 1963, 2013 and 2017, at only 14 d. In terms of the significance of the change trend, only the significant decrease in the number of precipitation days was significant at the 0.001 significance level, while the change trends of the other indicators were nonsignificant (Fig. 3).

#### Variability in extreme precipitation during the summer maize growing period

**Figure 4:**
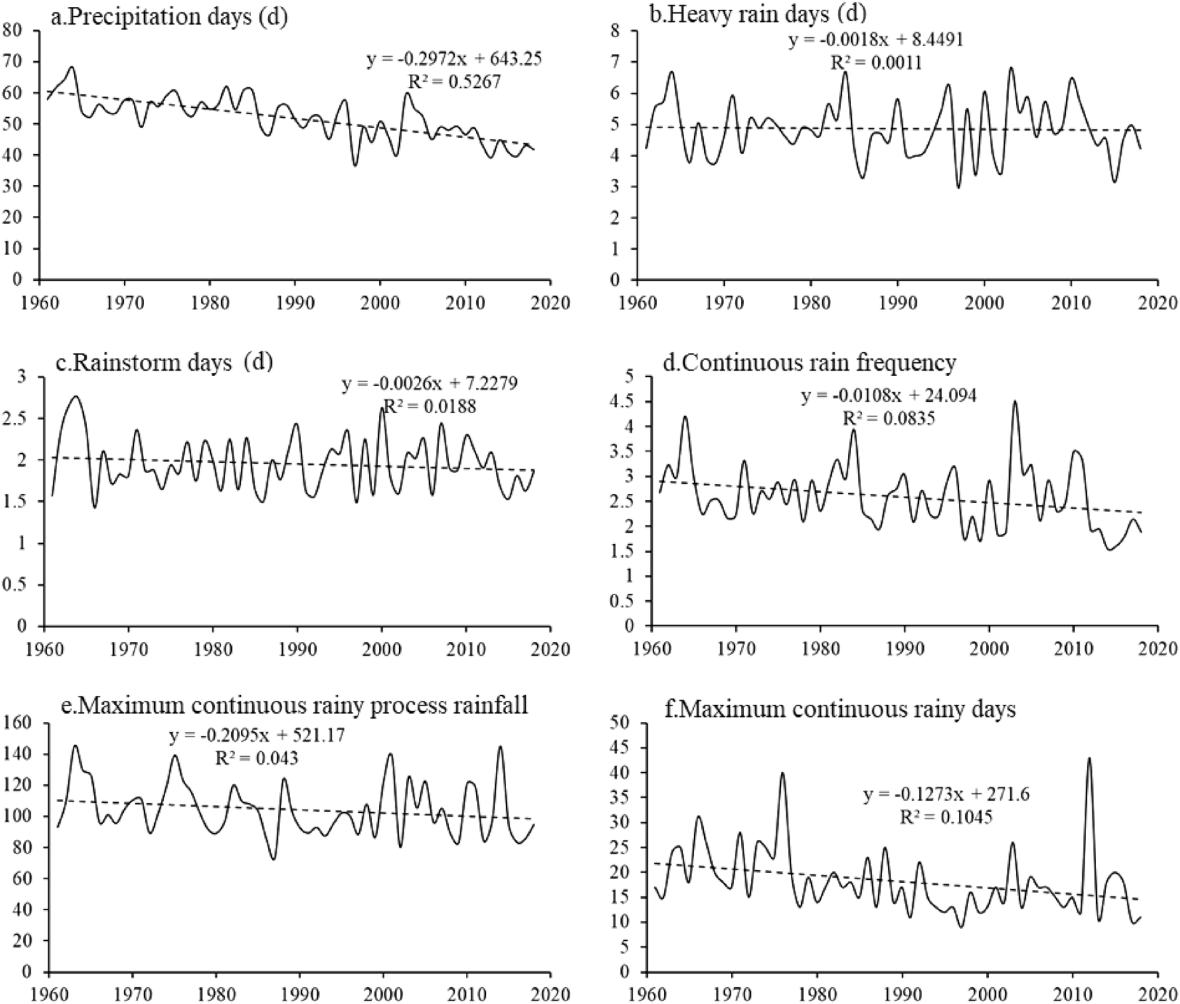
Extreme precipitation trends during the summer maize growing season from 1961 to 2018.

Figure 4 shows the interannual trend of the extreme precipitation index during the summer maize growing season in the north‒south transitional zone from 1961 to 2018. All the extreme precipitation indices decreased during the summer maize growing season (Fig. 4).

The number of rainy days decreased from 68 in 1963 to 41.8 in 2018. The year with the largest number of heavy rain days was 2003, with 6.8 d, while the year with the smallest number was 1997, with 2.97 d. The year with the largest number of rainstorm days was 1964, with 2.75 d, while the year with the smallest number was 1966, with 1.43 d. The year with the largest number of consecutive rainy days was 2003 (4.5), while the year with the smallest number was 2015 (1.6). The single maximum rainfall event occurred in 1963, with the total reaching 145.5 mm. The year with the greatest number of consecutive rainy days was 2013, with 43 days, followed by 1976, with 40 days, and 1997, with 9 days. In terms of the significance of the observed change, the changes in the number of precipitation days, frequency of continuous rain days and number of continuous rain days passed the 0.05 confidence test, and the decreasing trend was significant.

#### Temporal variability of drought events

**Figure 5:**
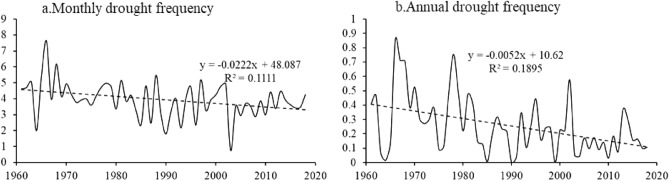
Trends of drought events from 1961–2018.

Figure 5 shows the interannual variability in drought events in the north‒south transitional zone from 1961 to 2018 at the monthly and annual scales. The monthly and annual drought frequencies decreased. At the monthly scale, the drought frequency in 1966 was the highest, at 7.63, while the drought frequency in 2003 was the lowest, at only 0.74. At the annual scale, the years with higher drought frequencies were 1966, 1978 and 2002, whereas there were no drought events in 1990 or 1999. In terms of the significance of the observed change, the changes in the monthly and annual drought frequencies passed the confidence tests at the 0.01 and 0.001 levels, respectively, indicating that the drought frequency decline at these two scales was significant (Fig. 5).

### Spatial distribution of major meteorological disasters in the north‒south transitional zone

To explore the disturbance caused by major meteorological disasters in the north‒south transitional zone, the spatial distributions of the changes in extreme precipitation and drought frequency at each meteorological station were analysed. Complex regions inconsistent with the overall change trend of meteorological disasters in the north‒south transitional zone were identified. This study could provide a basis for identifying the vulnerable areas of agricultural production in the north‒south transitional zone.

#### Spatial distribution of extreme precipitation variability during the winter wheat growing season

In terms of the trend in extreme precipitation in the north–south transitional zone during the winter wheat growing season, the number of precipitation days at most stations in northwest Shandong, southeast Hebei, southern Shanxi and northern Henan declined. The number of precipitation days at some stations in southern Henan and southern Gansu increased, but the upwards and downwards trends were not significant. The number of heavy rain days at most sites increased, and the sites with a significant increase were mainly distributed in Henan, Shaanxi and Gansu, while a few sites with a significant decrease occurred in the provinces of the north–south transitional zone.

**Figure 6:**
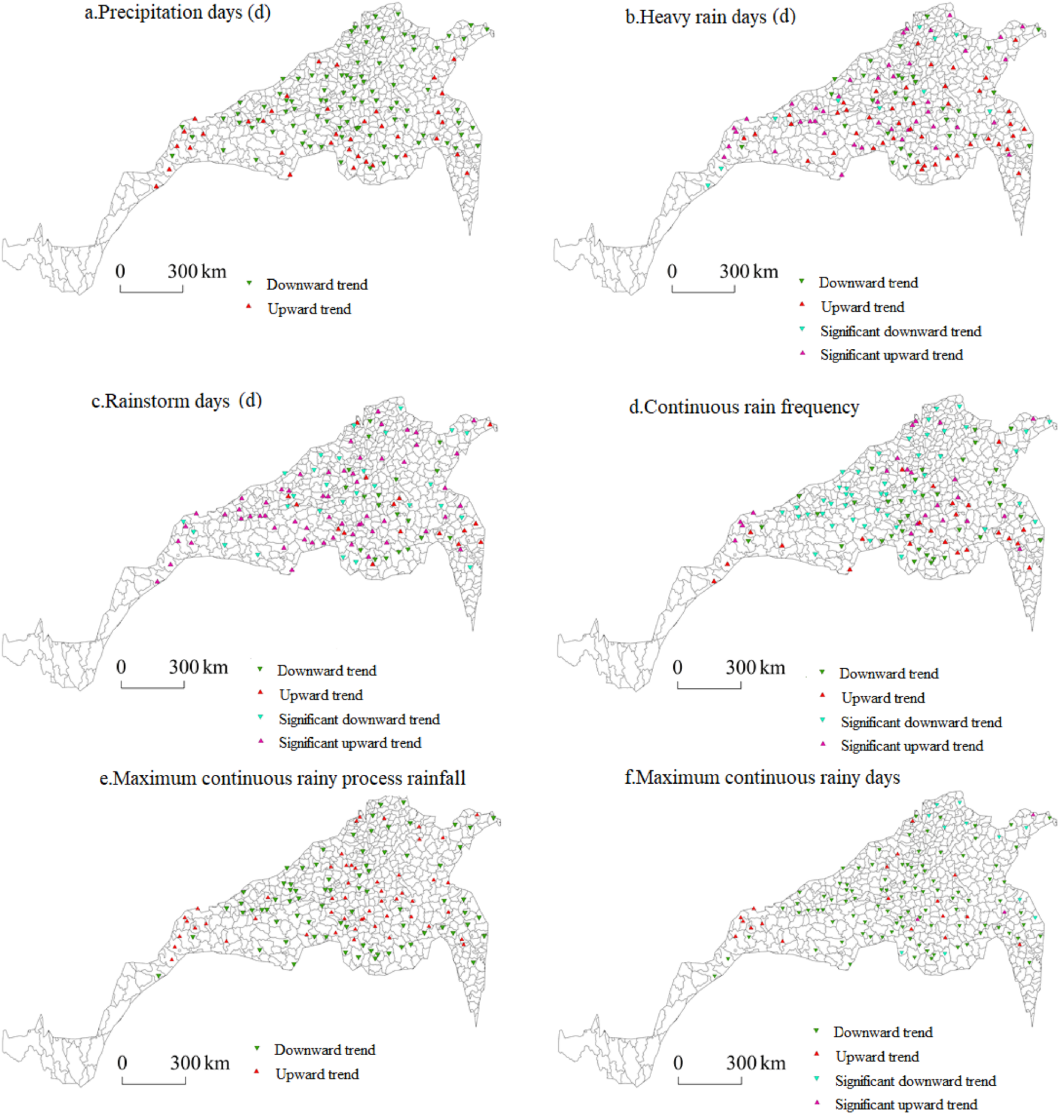
Spatial distribution of extreme precipitation during the winter wheat growing season.

The number of sites with an increasing number of rainstorm days exceeded that with a decreasing number of rainstorm days. The number of sites in central Shaanxi and western and central Henan increased, while the number of sites in southern Shanxi, northern Henan and southeastern Shandong decreased. The frequency of continuous rain decreased in most regions of the north–south transitional zone. The frequency of continuous rain increased at only a few sites in central Shaanxi, southern Shanxi and central and western Henan, while the other sites showed decreasing trends. The maximum process rainfall decreased at most of the stations in Hebei, Shaanxi, Shanxi, Jiangsu and Anhui and increased in southern Gansu, central and eastern Henan and southern Shandong. However, the observed increases and decreases were not significant. In the north–south transitional zone, the number of sites with the greatest number of continuous rainy days decreased more than the number of sites with an increase. Regionally, only one site in southern Gansu exhibited a decrease, while the other sites exhibited increases, and only two to four sites in the other provinces demonstrated increases. The stations with increasing numbers of precipitation days, heavy rain days and heavy rain days showed high spatial agreement with the typical regions, indicating that there was disaster disturbance resulting from extreme precipitation during the winter wheat growing season (Fig. 6).

#### Spatial distribution of extreme precipitation variability during the growing season of summer maize

The number of precipitation days at all stations in the north‒south transitional zone during the growing season of summer maize decreased, which is consistent with the overall temporal variability in the number of precipitation days. This indicated that precipitation decreased during the summer maize growing season in the north–south transitional zone, but the decline was not significant. The number of heavy rain days decreased in Shandong, Jiangsu and Shanxi and increased in Gansu. In other regions, the number of heavy rain days increased and decreased at the Sanmenxia and Wudu stations, respectively, while Qinyang and Jining showed significant decreases.

The stations with increases in the number of rainstorm days were mainly concentrated in central Shaanxi and southwest Henan, while the stations with decreases were largely concentrated in southeast Hebei and northwest Henan. All stations with an increase in Shaanxi exhibited significant changes, while only four stations in Henan indicated significant changes. The number of continuous rainy days in Hebei, Shandong and northern Henan significantly decreased, while the number of continuous rainy days in central Shaanxi, southern Shanxi and western Henan increased, but the observed change was not significant.

**Figure 7:**
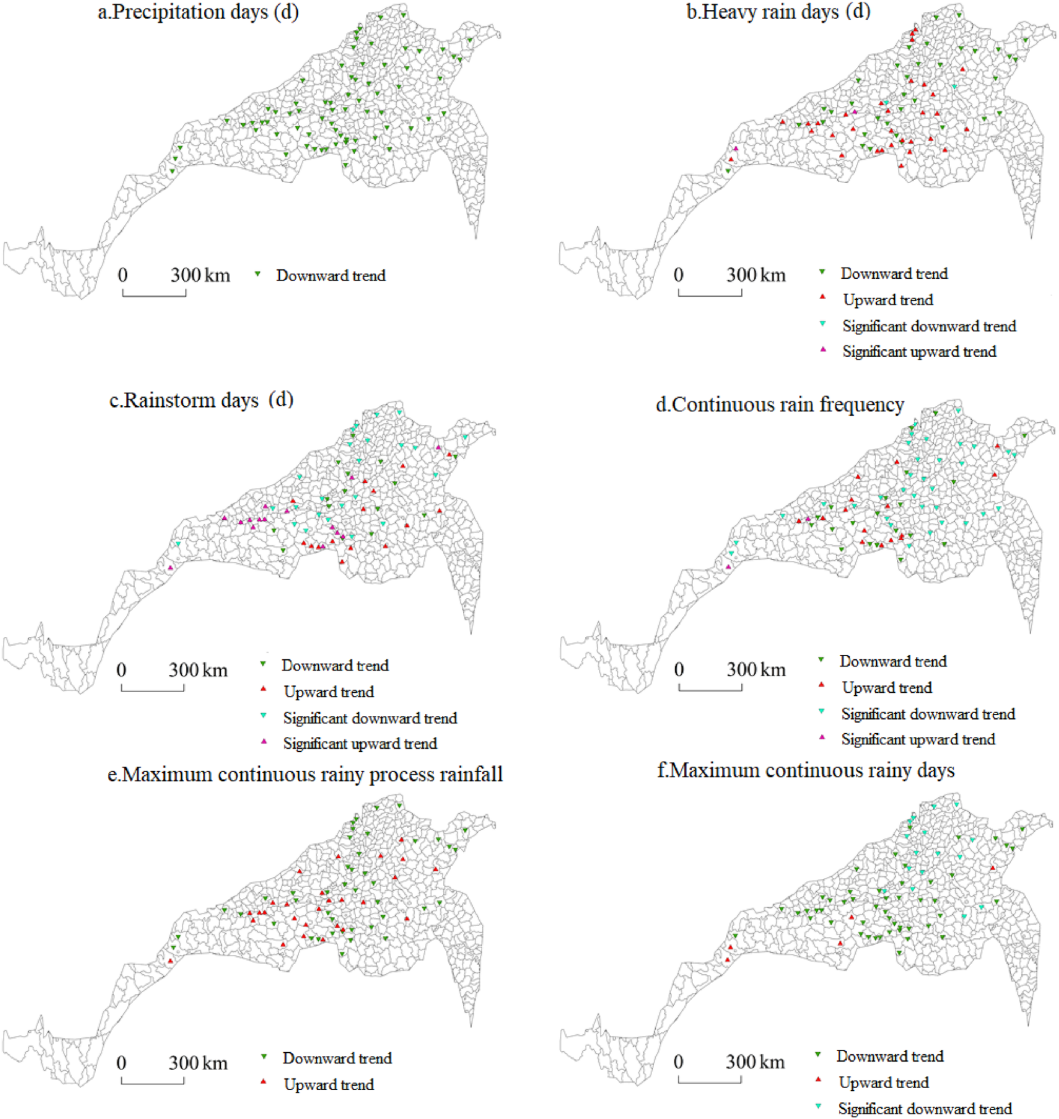
Spatial distribution of extreme precipitation changes during the growing season of summer maize.

Most of the stations in Hebei, eastern Shandong and central and northern Henan showed a decline in the maximum rainfall during continuous rain, while the stations in western Henan and central Shaanxi showed increases, but the changes were not significant. During the summer maize growing season in the north–south transitional zone, the largest number of consecutive rainy days at most stations decreased, and it increased at only one station each in Shandong, Gansu, Henan, Sichuan and Hubei. The largest number of consecutive rainy days significantly decreased, mainly in southeastern Hebei, northwestern Shandong and northern Henan. The number of rainy days, the number of heavy rain days, the frequency of continuous rain and the maximum amount of process rainfall during continuous rain showed high spatial agreement with the typical regions, indicating that disaster disturbance due to extreme precipitation also occurred during the summer maize growing period in the typical regions (Fig. 7).

#### Spatial distribution of drought temporal variability in the north–south transitional zone

**Figure 8:**
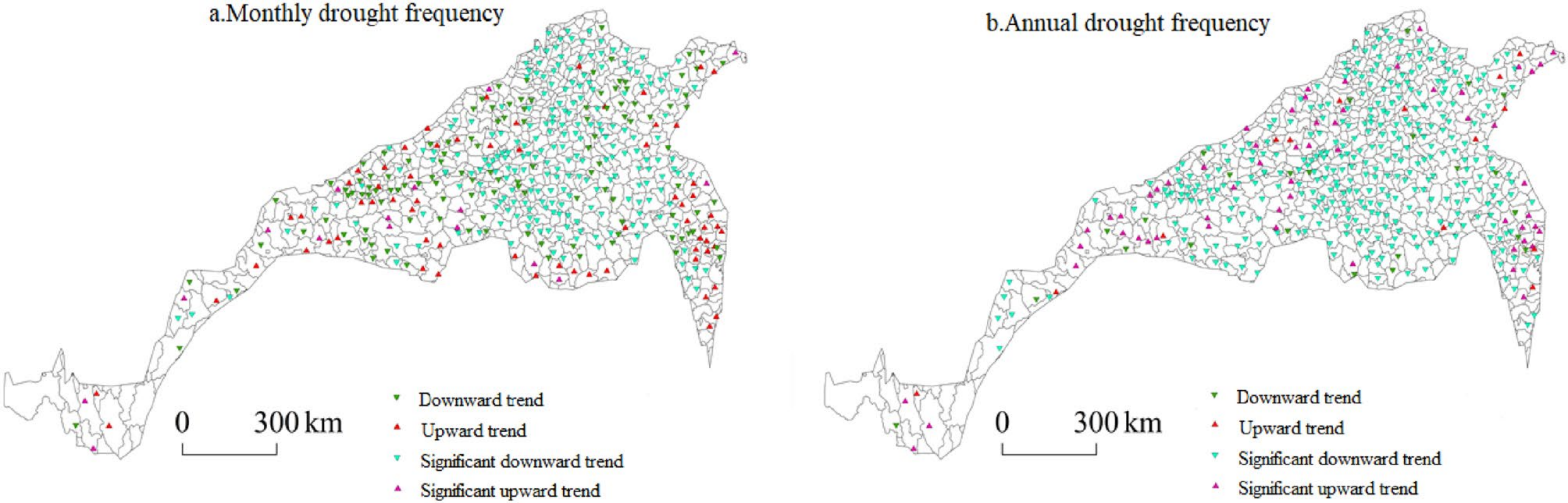
Spatial distribution of the drought variability.

The drought grades at the meteorological stations in the north–south transitional zone were determined according to the criteria for the classification of meteorological drought grades, and the total frequencies of light drought, moderate drought, severe drought and extreme drought at the monthly and annual scales were obtained. A spatial distribution map of the drought event variability in the north–south transitional zone was created, and the change trend at the meteorological stations from 1961 to 2018 was observed. In terms of the monthly drought variability, the drought frequency at most stations in the eastern and middle parts of the north–south transitional zone in Hebei, Shandong, Henan, Anhui and Shanxi significantly decreased, while in central and southern Shaanxi and southeast Jiangsu, it increased, and Shaanxi exhibited the most significant increase. In terms of the annual drought variability, the sites with significant decreases were mainly concentrated in Hebei, Anhui, Shandong, Henan and Jiangsu, while the sites with significant increases were largely distributed in southeast Shandong, southeast Jiangsu, southern Shanxi and the junction of Gansu and Shaanxi. The sites with increasing monthly and annual drought levels spatially overlapped with the typical regions, indicating that winter wheat and summer maize in the typical regions of the north‒south transitional zone were disturbed by drought disasters at different scales (Fig. 8).

### Spatial distribution characteristics of the major meteorological disasters in the north–south transitional zone

#### Spatial distribution of extreme precipitation during the winter wheat growing season

**Figure 9:**
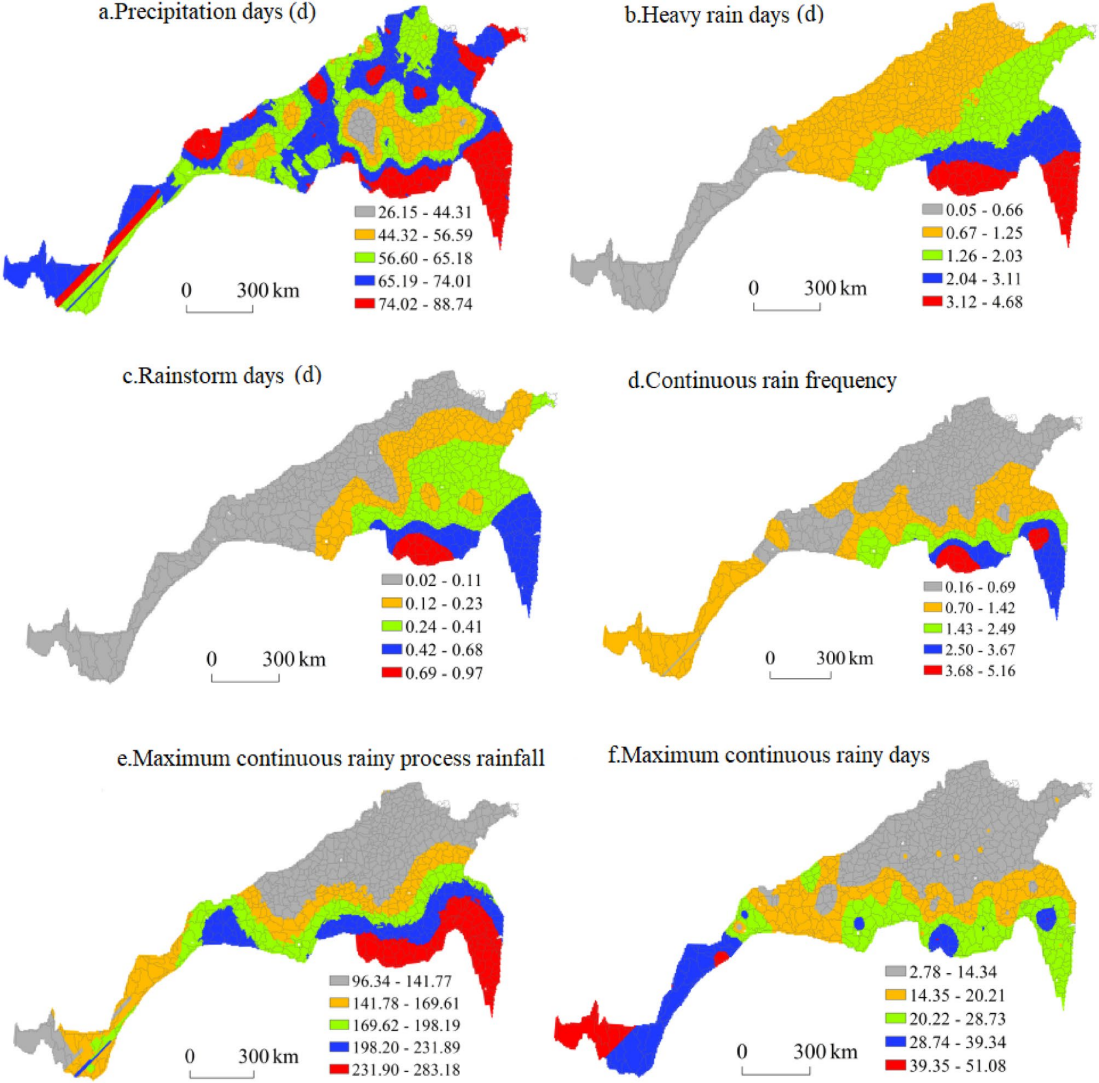
Spatial distribution of extreme precipitation during the winter wheat growing season.

Figure 9 shows the spatial distribution characteristics of each extreme precipitation index during the winter wheat growing season. The regions with the greatest number of precipitation days were mainly concentrated in southeastern Jiangsu Province, including Shouxian, Changfeng, Huoqiu and Lu’an in Anhui Province; Luoshan, Xinxian, Chengcheng and Gushi in Henan Province; Yantai, Muping, Wendeng, Gaomi and Anqiu in Shandong Province; Li County; Tianshui and Hui County in Gansu Province; and Hongdong in southern Shanxi Province. The regions with more precipitation days were primarily concentrated in central Shandong, southern Hebei, and southern and southeastern Shaanxi. The regions with a moderate number, small number and smallest number of precipitation days were mainly concentrated in northwest Jiangsu, northern Anhui, central and northern Henan and southern Shaanxi, respectively, and the number of precipitation days increased from the centre towards the periphery, among which Yuzhou and Ruzhou in Henan were the centres of low precipitation days. The largest number of precipitation days in the region reached 88.74 days, while the smallest number of precipitation days reached only 26.15 days, indicating that the precipitation distribution was uneven and that a large difference occurred in the north–south transitional zone during the winter wheat growing season.

The number of heavy rain days during the winter wheat growing season decreased from southeast to northwest. The region with the greatest number of heavy rain days was concentrated in the southern part of the eastern section of the north–south transitional zone. These provinces included southern Jiangsu, western Anhui and southern Henan, followed by northern Jiangsu, northern Anhui and central and southern Henan. The regions with a moderate number of heavy rain days were mainly concentrated in southeast Shandong, central Henan and northwestern Hubei. The regions with the smallest number of heavy rain days were southern Gansu and central Sichuan. The largest number of rainy days reached 4.68, and the smallest number of heavy rain days was 0.05.

The overall spatial distribution characteristics of rainy days during the winter wheat growing season were similar to those of heavy rain days, showing a decreasing spatial distribution from southeast to northwest. This finding is consistent with the spatial distribution characteristics of precipitation in China. The regions with the greatest number of rainstorm days were Luoshan, Xinyang, Shangcheng and Gushi in southern Henan Province, central and southern Jiangsu Province, western Anhui Province and central and southern Henan Province, respectively. The regions with moderate and small numbers of rainstorm days occurred mainly in northern Jiangsu Province, central and southern Shandong Province and central and eastern Henan Province. The regions with the fewest rainstorm days included northern Shandong, northeastern Hebei and Shanxi, central and southern Shaanxi, southern Gansu and central Sichuan. The largest number of heavy rain days in the area reached 0.97 days, and the smallest number of heavy rain days reached 0.02 days.

The regions with the highest frequency of continuous rain were mainly concentrated in Gaoyou in Jiangsu Province and Xinyang, Luoshan, Shangcheng and Xinxian in Henan Province. Regions with higher continuous rain frequencies were located in central and southern Jiangsu Province and western Anhui Province. The regions with medium and low frequencies of continuous rain were concentrated in northern Jiangsu Province, northern Anhui Province, southern Shandong Province, central and western Henan Province, southern Shaanxi Province and central Sichuan Province. The regions with the smallest number of consecutive rainy days occurred in northeast Shandong, southeast Hebei, south Shanxi, central and northern Henan and southwest Shaanxi. The maximum number of consecutive rainy days was 5.16 days, and the smallest number was 0.16 days.

The maximum process rainfall during the winter wheat growing season was concentrated in southwestern Jiangsu, central Anhui and southern Henan. Higher rainfall was mainly concentrated in northeastern Jiangsu and Anhui and central and southern Henan, and medium and low rainfall levels were mainly concentrated in southern Shandong, northern Anhui, central and southern Shaanxi, northwestern Hebei and southern Gansu. The low-value areas were largely concentrated in northeast Shandong, southeast Hebei, south Shanxi, north Henan and central Shaanxi. The maximum continuous rainfall reached 283.18 mm, and the lowest rainfall level reached 96.34 mm.

The region with the greatest number of consecutive rainy days during the winter wheat growing season was located in eastern Xizang Province. The regions with the highest values were located in Gaoyou in Jiangsu Province, Xinyang and Chengcheng in Henan Province and Yunxi in Hubei Province. The regions with medium and low values were concentrated in northern central Jiangsu and Anhui Provinces, southwestern Henan Province and southern central Shaanxi Province, and the regions with the lowest values were located in Shandong Province, southeastern Hebei Province, southeastern Shanxi Province and northeastern Henan Province. The maximum number of consecutive rainy days was 51.08 days, and the minimum number was 2.78 days.

#### Spatial distribution of extreme precipitation during the summer maize growing season

**Figure 10:**
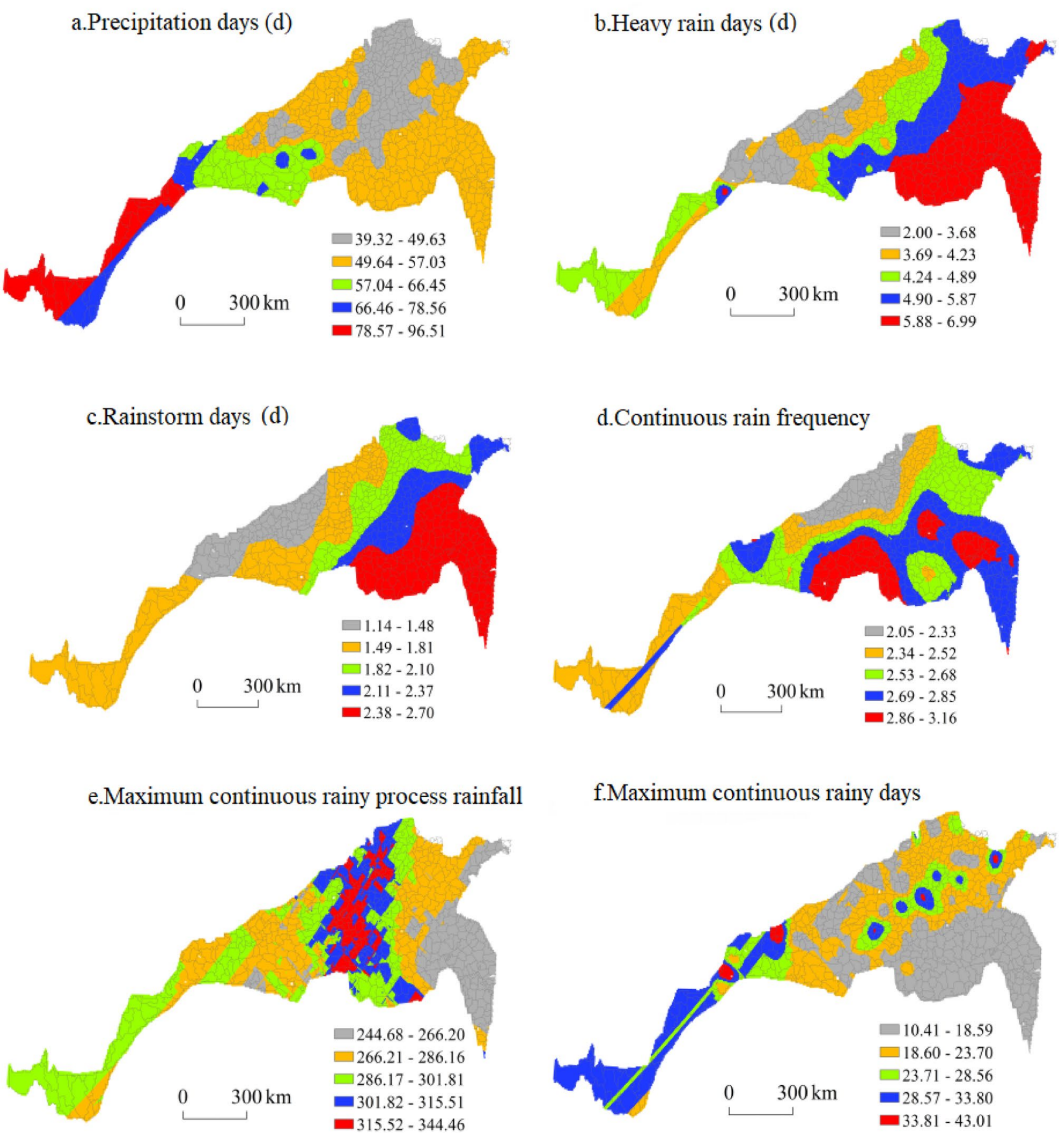
Spatial distribution of extreme precipitation during the summer maize growing season.

Figure 10 shows the spatial distribution characteristics of each extreme precipitation index during the summer maize growing season. The regions with the largest and largest numbers of precipitation days were mainly concentrated in central Sichuan and southern Gansu, and there were sporadic distributions in Danfeng and Ankang in southern Shaanxi and Lushi in Henan. The areas with a moderate number of precipitation days were mostly concentrated in southern Shaanxi, western Henan and northwestern Hubei. Areas with a small number of precipitation days were concentrated in southeastern Shandong, northwestern Jiangsu, southern Shanxi, southern and northwestern Henan and central Shaanxi. The areas with the smallest number of precipitation days were northwestern Shandong, southeastern Hebei, north-central Henan and east-central Shaanxi. The largest number of precipitation days in the area reached 96.51 days, while the smallest number reached only 39.32 days, indicating that the precipitation distribution during the summer maize growing season in the north–south transitional zone greatly differed from that during the winter wheat growing season.

The number of heavy rain days during the summer maize growing season decreased from southeast to northwest, and the region with the greatest number of heavy rain days was concentrated in the southeast of the eastern section of the north–south transitional zone, including southern Jiangsu, northern Anhui, southern Henan and southern Shandong. This region was followed by northeast Shandong, southeast Shaanxi, northwest Hubei, east Hebei and southeast Henan. The regions with moderate and small numbers of heavy rain days were largely concentrated in southeastern Hebei, Shanxi and Shaanxi, northwestern Henan and central Sichuan, and the regions with the smallest number of heavy rain days occurred in southwestern Shanxi, central Shaanxi and southern Gansu. The largest number of heavy rain days in the area was 6.99 days, and the smallest number of heavy rain days was 2.00 days.

Southern Jiangsu, northern Anhui, southern Henan and southern Shandong were the regions with the most rainstorm days during the summer maize growing season, while eastern Henan, central Shandong, eastern and eastern Hebei were the regions with many rainstorm days. The regions with a moderate or small number of rainstorm days were mainly concentrated in southeastern Hebei, northwestern Shandong, southeastern Shanxi, southern Shaanxi, northwestern Henan and central Sichuan. The regions with the smallest number of rainstorm days occurred in southwestern Shanxi, central Shaanxi and southern Gansu. The largest number of heavy rain days in the area reached 2.70 days, and the smallest number of heavy rain days reached 1.14 days.

The areas with a high frequency of continuous rain during the summer maize growing season were mainly concentrated in northwestern Jiangsu, southern Shandong, southwestern Henan and northwestern Hubei. The areas with a high frequency of continuous rain during the summer maize growing season were located in southeast Jiangsu, east Anhui, east Henan, northeast and southwest Shandong, and southwest and southeast Shaanxi. The regions with a moderate continuous rain frequency were concentrated in central Shandong, northwest Anhui, south-central Shaanxi, south-central Henan and southwestern Gansu. The regions with a low frequency and the lowest frequency of continuous rain were southeastern Hebei, southeastern Shanxi, central Shaanxi and central Sichuan. The highest frequency of continuous rain was 3.16 days, and the lowest frequency reached 2.05 days.

The maximum rainfall during continuous rain in the summer maize growing season exhibited a spatial distribution that decreased from a central high-value area towards both sides. The regions with high and highest values of the maximum process rainfall were concentrated in the middle of the north–south transitional zone, including southeast Hebei, north-central Henan and western Anhui. The medium- and low-value regions covered northeastern Shandong, northwestern Anhui, south-central Shaanxi, south-central Gansu and central Sichuan, while the low-value areas were mainly concentrated in northwestern Jiangsu, southeastern coastal Shandong and northeastern Anhui. The maximum continuous rainfall during the summer maize growing season was 344.46 mm, and the minimum rainfall was 244.68 mm.

In the eastern, middle and western parts of the north–south transitional zone, the maximum number of continuous rainy days during the summer maize growing season was the largest at the centre, and spatially, the number of rainy days decreased from the centre of the high-value area towards the periphery. The high-value and second-highest-value areas in the eastern section occurred in Weifang, Tai’an, Liaocheng and Heze in Shandong Province. High-value and second-highest-value areas in the middle section occurred in Luanchuan, Puyang and Xinxiang in Henan Province. High-value and second-highest-value areas in the western section were distributed in Fengxiang and Baoji in Shaanxi Province and Wenxian in Sichuan Province. The medium- and low-value areas occurred at the periphery of the high-value areas, mainly including southeast and central Shandong, southeast Hebei, southeast Shanxi, central and southern Shaanxi and northwest Henan, while the lowest-value areas mainly occurred in northwest Jiangsu, north Anhui and southeast Henan. Moreover, there were sporadic distributions in Shandong, Hebei, Shanxi and Shaanxi. The maximum number of consecutive rainy days was 43.01 days, and the minimum number reached 10.41 days.

#### Spatial distribution of drought frequency based on SPEI_1_

**Figure 11:**
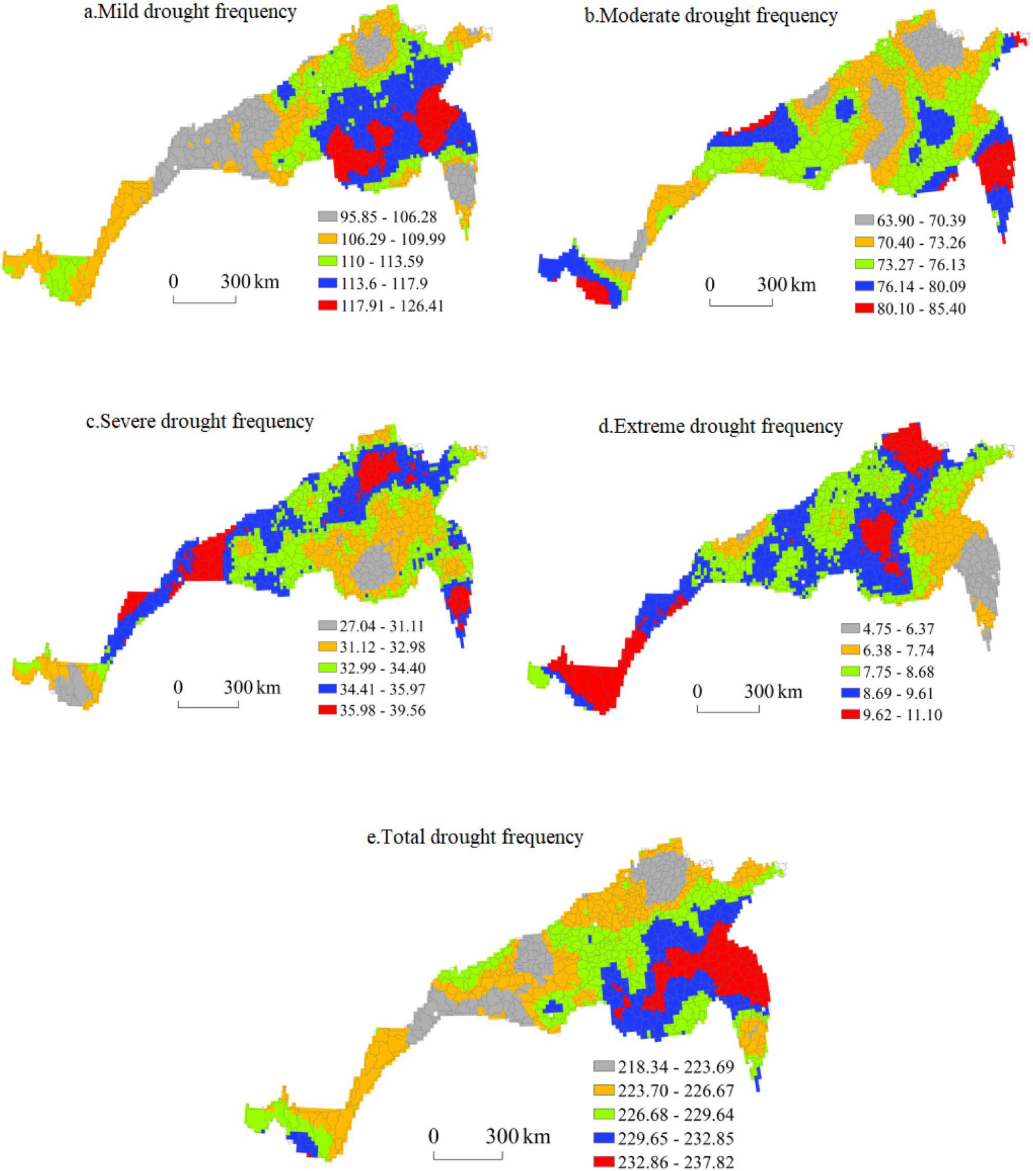
Spatial distribution of the drought frequency based on the SPEI_1_.

Figure 11 shows the spatial distribution of the drought frequency in the north–south transitional zone based on the SPEI_1_. The areas with a high frequency of light drought were mainly concentrated in northeastern Jiangsu and southeastern Henan; the areas with the second-highest values largely occurred in central Jiangsu, southern Shandong, northeastern Anhui and central Henan; and the areas with moderate and low drought frequencies were mostly concentrated in northern Shandong, southeastern Shanxi, western Henan, central Anhui, central Sichuan and northwestern Hubei. The regions with the lowest frequency of light drought were concentrated in central and southern Shaanxi and southern Gansu. The highest frequency of light drought in the region reached 126.41, and the lowest frequency of light drought reached 95.85.

The high-value and second-highest-value areas with a medium drought frequency were mainly concentrated in central Jiangsu, central Anhui, central Shaanxi, northern Yunnan and the junction of Shandong, Anhui and Jiangsu Provinces, while the medium- and low-frequency areas occurred in southeast Shandong, northwest Henan, northeast Jiangsu, southeast Hebei, southeast Shanxi, central Anhui, southeast Shaanxi, central Sichuan and northwest Hubei. The regions with the lowest frequency of medium drought were concentrated in northeastern Shandong and central Henan. In the region, the highest frequency of moderate drought was 85.4 times, and the lowest frequency of moderate drought reached 63.9 times.

The high-value areas of severe drought frequency were mainly concentrated in southern Jiangsu, northwest Shandong, southwest Shaanxi and central Sichuan; the second-highest-value areas largely occurred in northeastern Shandong, northern Henan and central Shaanxi; and the medium- and low-frequency areas were mostly concentrated in northwestern Jiangsu, northeast Anhui, southern Shandong, central Henan, southeast Shanxi and central and southern Shaanxi. The regions with the lowest frequency of severe drought were northwestern Anhui, northern Yunnan and southeastern Henan. In the region, the highest frequency of severe drought reached 39.56, and the lowest frequency of severe drought reached 27.04.

The areas with high values of extreme drought frequency were mainly concentrated in northeast Shandong, southeast Hebei, east Henan and central Sichuan; the areas with the second-highest values and medium values were mainly distributed in southeast Shandong, southwest Hebei, southeast Shanxi, south central Shaanxi and west central Henan; and fewer areas were mainly concentrated in northeast Jiangsu, northeast Anhui and southwest Shaanxi. In the region with the highest frequency of extreme drought, it occurred 11.1 times, and in the region with the lowest frequency of extreme drought, it occurred 4.75 times.

The regions with high total drought frequencies were mainly concentrated in northeast Jiangsu, southeast Henan and north Anhui; the regions with the second-highest and moderate drought frequencies largely occurred in Anhui, central Henan and southwest Shaanxi; and the regions with low and lowest total drought frequencies were mostly concentrated in northeast Shandong, southeast Hebei, southeast Shanxi, central and south Shaanxi, west Henan and central Sichuan. The highest total drought frequency in the region was 237.82 times, and the lowest total drought frequency reached 218.34 times.

#### Spatial distribution of the drought frequency based on the SPEI_12_

**Figure 12:**
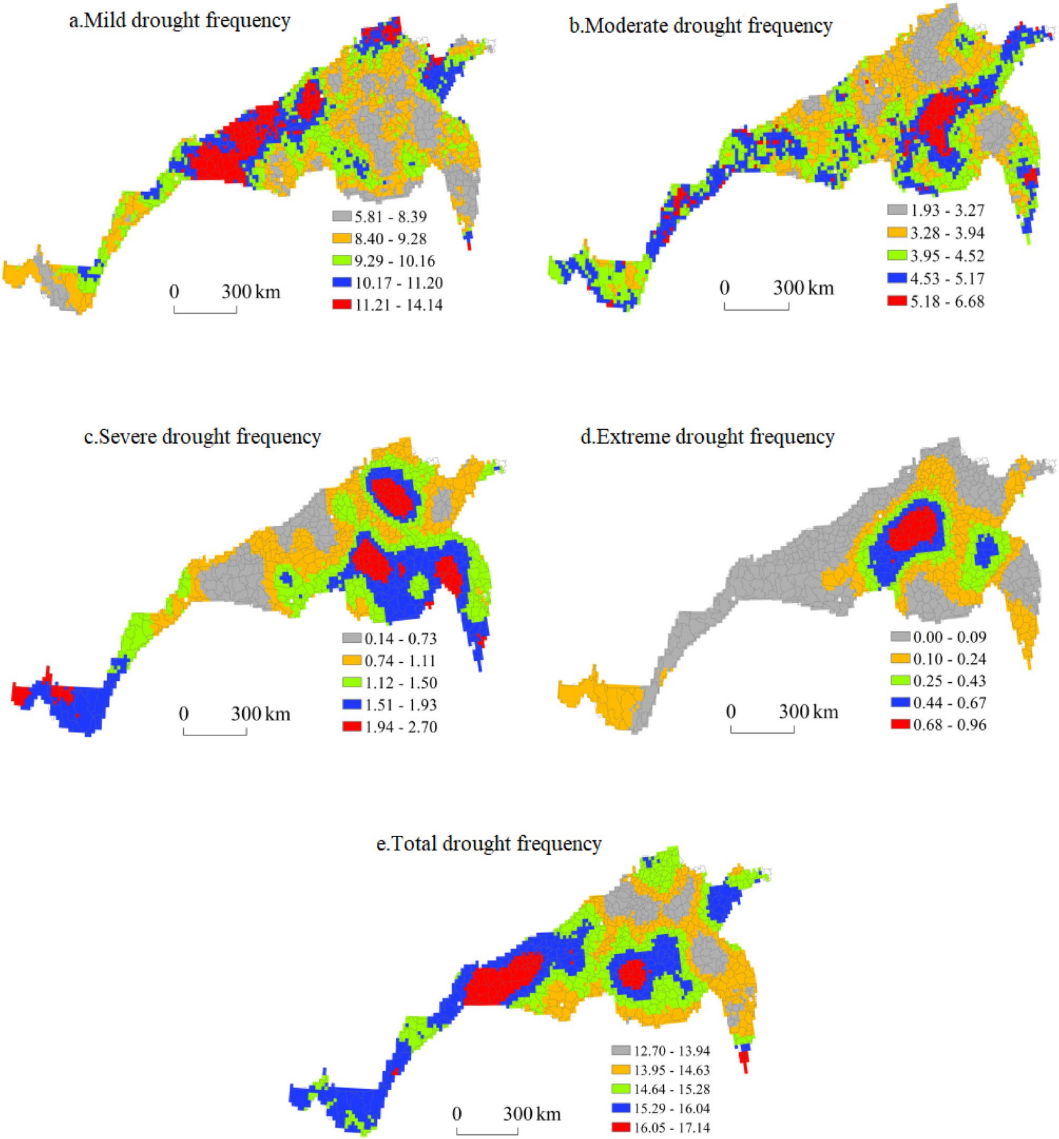
Spatial distribution of the drought frequency based on the SPEI_12_.

Figure 12 shows the spatial distribution of the drought frequency in the north–south transitional zone based on the SPEI_12_. The areas with the highest and second-highest frequencies of light drought were mainly concentrated in eastern Hebei, southeastern Shanxi, northeastern Shandong and southeastern Shaanxi, and the areas with moderate and low frequencies of light drought occurred in Henan, northern Jiangsu, northwestern Shandong, southeastern Hebei, central Sichuan and northwestern Hubei. The areas with the lowest frequency of light drought were concentrated in southern Jiangsu, central and southern Shandong and northwestern Anhui. In the region, the highest frequency of light drought reached 14.14, and the lowest frequency of light drought was 5.81.

The highest and second-highest frequencies of medium drought were mainly observed in southeast Shandong, eastern Henan, southern Jiangsu, northwest Anhui, southwest Shaanxi and central Sichuan, while the medium- and low-frequency areas were largely concentrated in central Jiangsu, northeast Anhui, northeast Shandong, southwest Hebei, southeast Shanxi and central Shaanxi. The regions with the lowest frequency of medium drought were concentrated in western Shandong, northern Jiangsu and southeastern Hebei. In the region, the highest frequency of moderate drought reached 6.68, and the lowest frequency of moderate drought was 1.93.

The highest and second-highest frequencies of severe drought were mainly observed in northwest Jiangsu, southeast Henan, west Shandong, south Hebei and north Yunnan, while the medium- and low-value areas were primarily concentrated in northeast Jiangsu, southeast Shandong, southeast Hebei, northwest Henan, southeast Shanxi, central Sichuan and southeast Shaanxi. The regions with the lowest frequency of severe drought were concentrated in central Shandong, central Shanxi and southwestern Shaanxi. In the region, the highest frequency of severe drought reached 2.70 times, while the lowest frequency of severe drought reached 0.14 times.

The areas with the highest and second-highest frequencies of extreme drought were mainly concentrated in northeast Henan and northeast Jiangsu; the areas with medium and low frequencies were largely concentrated in southwest Shandong, northern Anhui, southern Hebei, southern Shaanxi, western Henan and southeastern Shaanxi; and the areas with the lowest frequency of extreme drought were concentrated in central and southern Shaanxi, eastern Jiangsu, northern Shandong, southeast Shanxi, southern Gansu and central Sichuan. In the region, the highest frequency of extreme drought was 0.96 times, while the lowest frequency of extreme drought was zero.

The regions with the highest and second-highest total drought frequencies were mainly concentrated in central and eastern Henan, southeast Shaanxi, southeast Shandong and central Sichuan, while the regions with medium and low total drought frequencies were largely concentrated in central Shandong, eastern Jiangsu, northern Anhui, northwest Henan, southeast Hebei, southeast Shanxi and northwest Hebei. The regions with the lowest total drought frequency were concentrated in northwestern Jiangsu, central Shandong and southern Hebei. In the region, the highest total drought frequency reached 0.30, while the lowest total drought frequency reached 0.22.

### Meteorological disaster stress in China’s north‒south transitional zone

**Figure 13:**
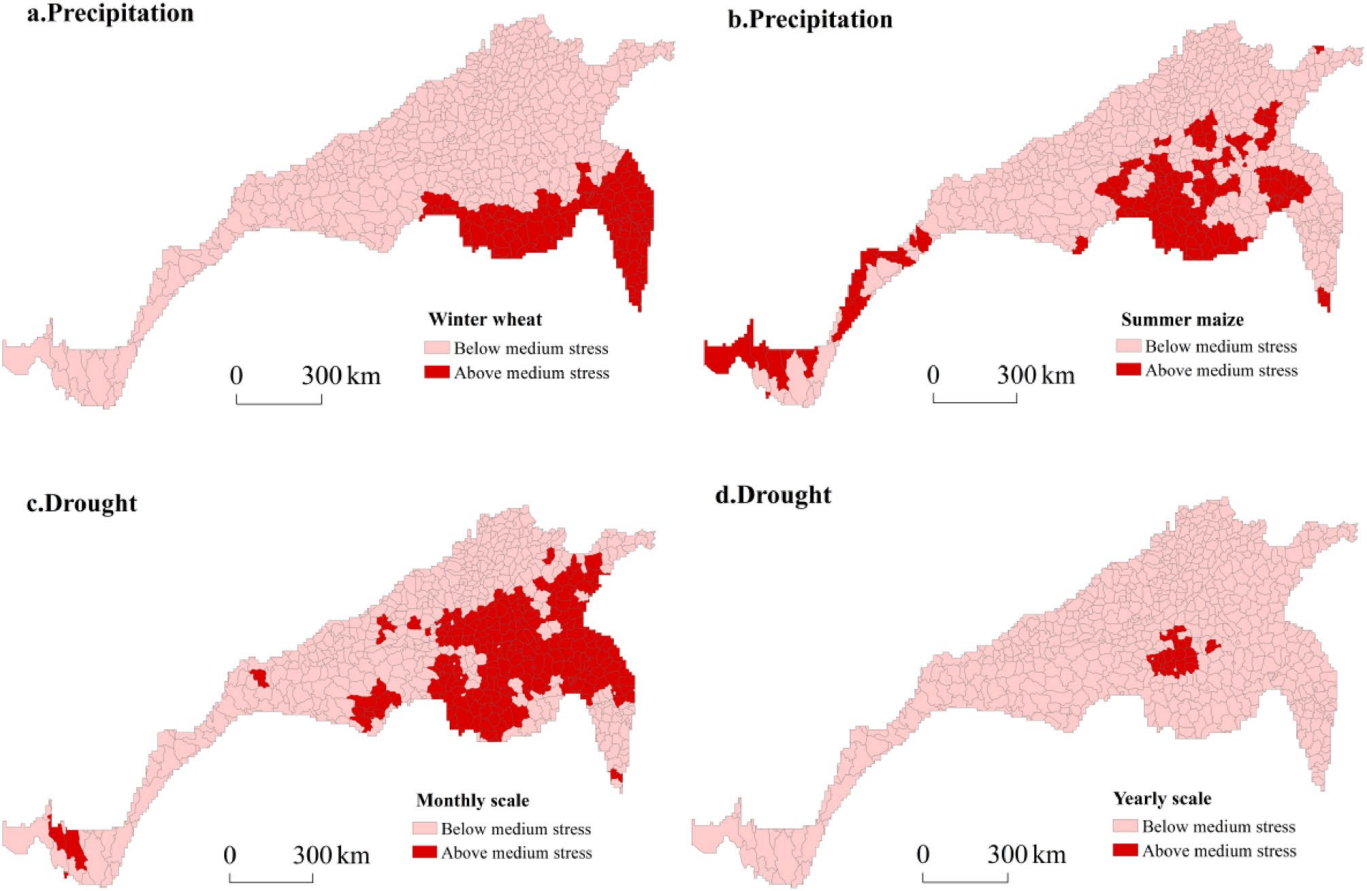
Spatial distribution of extreme precipitation and drought stress.

To accurately identify the risks of precipitation and drought stress faced by winter wheat and summer maize within each county of the research area, ArcGIS zoning statistics were utilized to extract regions at the top three levels of extreme precipitation during the winter wheat growing season, extreme precipitation during the summer maize growing season, monthly scale drought, and annual-scale drought. These regions were subsequently visualized in a single map. Figure 13 shows a spatial distribution map, where the red areas denote regions with medium or higher levels of stress according to all indicators, while the pink areas indicate regions with lower levels of stress based on all indicators. Figure 13a reveals a concentrated spatial distribution of regions with medium or high levels of extreme precipitation during the winter wheat growing season. These regions are primarily located in southern Henan Province, central Anhui Province, central and southwestern Jiangsu Province, and northwestern Zhejiang Province—all areas that are vulnerable to disruptions in winter wheat growth due to climate-induced precipitation instability. The spatial distribution range of the extreme precipitation indicators during the summer maize growth period in areas with moderate stress and above is shown in Fig. 13b. These indicators were observed in southern Tibet, central Sichuan, central–southern Henan, northern Anhui, southern Shandong, and northwestern Jiangsu. Notably, Henan Province exhibited a wide distribution range, and increased attention should be given to preventing extreme precipitation disasters during the summer maize growth period in this region. Figure 13c shows the spatial distribution range of medium to severe drought-prone areas in the north‒south transitional zone of China at the monthly scale, which are mainly concentrated in Henan, Shandong, Anhui, and Jiangsu Provinces. There were also scattered distributions in Hubei, Gansu, Yunnan, and Tibet. On an annual scale, medium to severe drought-prone areas occur only in Henan Province.

**Figure 14:**
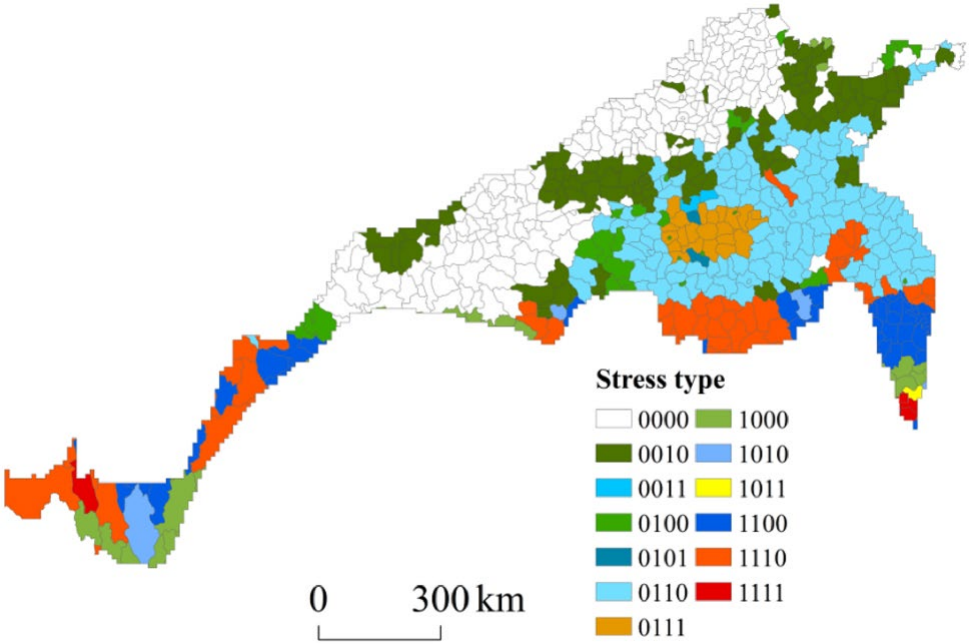
Types of climatic disaster stress in agricultural production.

To evaluate the magnitude of extreme precipitation and drought disturbances in the north–south transitional zone of China, a value of 1 was assigned to indicate moderate or higher stress, while a value of 0 was assigned otherwise. The climate disaster disturbance conditions in each county were categorized into 13 types. Figure 14 shows the results, while the definitions and proportions are listed in Table 4. The findings suggested that approximately 20.25% of the total number of counties (129) were exposed to both extreme precipitation (summer maize) and monthly scale drought stress. Additionally, approximately 15.54% of the counties (99) experienced monthly drought stress only, while 10.68% of the counties (68) experienced all three types of drought stress simultaneously. Only three regions concurrently experienced threats resulting from four types of stresses, accounting for approximately 0.47% of the total amount. Overall, approximately 63.58% of China’s north‒south transitional zone was characterized by moderate or higher stress levels, primarily concentrated along its southern boundary and central core area, and nearly 39.5% of the counties suffered two or more types of disaster stresses.

**Table 4:** Definition of the stress type code.

Stress type code	Definition	Number	Proportion (%)
0000	No medium or higher stress	232	36.42
0010	Monthly-scale drought	99	15.54
0011	Monthly-scale drought Yearly-scale drought	2	0.31
0100	Extreme precipitation (summer maize)	27	4.24
0101	Extreme precipitation (summer maize) Yearly-scale drought	2	0.31
0110	Extreme precipitation (summer maize) Monthly-scale drought	129	20.25
0111	Extreme precipitation (summer maize) Monthly-scale drought Yearly-scale drought	21	3.30
1000	Extreme precipitation (winter wheat)	27	4.24
1010	Extreme precipitation (winter wheat) Monthly-scale drought	4	0.63
1011	Extreme precipitation (winter wheat) Monthly-scale drought Yearly-scale drought	1	0.16
1100	Extreme precipitation (winter wheat) Extreme precipitation (summer maize)	43	6.75
1110	Extreme precipitation (winter wheat) Extreme precipitation (summer maize) Monthly-scale drought	47	7.38
1111	Extreme precipitation (winter wheat) Extreme precipitation (summer maize) Monthly-scale drought Yearly-scale drought	3	0.47
Total		637	100

## Conclusion

1. Under climate change, agricultural production in the north–south transitional zone has been disturbed to different degrees by meteorological disasters such as extreme precipitation and drought. In terms of the overall temporal variation, the degrees of extreme precipitation and drought stress faced by agricultural production in the region are decreasing. However, the temporal variation at each station in the north–south transitional zone was not completely consistent with the overall trend, and both increasing and decreasing trends were observed. The sites with an increase overlapped with the typical regions of the north–south transitional zone to varying degrees, indicating that the typical regions were not only theoretical potential risk areas under climate change but also suffered from meteorological disaster disturbances.
2. The distribution of precipitation during the winter wheat growth period in the south‒north transitional zone was uneven and varied significantly. The number of heavy rain days and rainstorm days exhibited a decreasing spatial pattern from southeast to northwest, which agrees with the overall precipitation distribution in China. High values of extreme precipitation indices during the winter wheat growth period were mainly concentrated in the southern part of the eastern section of the north‒south transitional zone. The precipitation distribution during the summer maize growth period significantly differed, with the highest amount of heavy rain and the greatest number of rainstorm days occurring in the southeastern part of the north‒south transitional zone. The spatial distribution of the drought frequency in the north–south transitional zone, as indicated by the SPEI_1_, showed that the areas with high total drought frequencies were mainly concentrated in northeast Jiangsu, southeast Henan, and north Anhui, which primarily experienced light drought. The central part of Jiangsu Province exhibited a high incidence of moderate drought, while southern Jiangsu Province and southwestern Shaanxi Province were prone to severe drought. Additionally, southeastern Hebei and eastern Henan were identified as areas with a high incidence of extreme drought. Finally, the central region of Sichuan Province was characterized by both severe and extreme drought. Considering the SPEI_12_-based spatial distribution of the drought frequency in the north–south transitional zone, the areas with a high total drought frequency were mainly concentrated in central and eastern Henan, southeast Shaanxi, southeast Shandong, and central Sichuan, which primarily experienced light to moderate drought. The northwestern part of Jiangsu, the southern part of Hebei, and the western part of Shandong were regions with high incidences of severe drought, while the eastern part of Henan was an area with high incidences of both severe and extreme drought.
3. High-value areas of extreme precipitation and drought disturbance in the north–south transitional zone overlapped with those at the edge of the transitional zone to varying degrees. Approximately 63.58% of the north–south transitional zone of China was characterized by moderate or high stress levels, primarily concentrated along its southern boundary and central core area, and nearly 39.5% of all counties suffered from two or more types of disaster stresses.

## Discussion

This study focused on analysing the temporal variation and spatial distributions of extreme precipitation and drought indicators during the winter wheat and summer maize growing periods, attempted to determine the temporal and distribution patterns of extreme precipitation and drought, and aimed to accurately identify the disaster stress levels and stress types faced by counties in the north–south transitional zone of China. The research findings are generally consistent with those of previous studies. In terms of the variation trend, during the winter wheat and summer maize growing periods, the degree of extreme precipitation and drought stress faced by agricultural production in the region decreased; this conclusion is consistent with the results of Xu, Zhai, Zhang et al.^33–35^. In terms of specific regions, the research findings of this article are consistent with the records in yearbooks and previous studies on areas prone to frequent continuous rainy disasters. For instance, Fuyang, Anhui Province, has experienced multiple severe continuous rainy disasters during the summer harvest period^36,37^. Similarly, Zhumadian, Henan Province, has experienced frequent continuous rainy disasters at the mid-to-late growth stages of winter wheat^38^. There are still areas for improvement in this study. For instance, the determination of precipitation statistics based on the average harvest dates of winter wheat and summer maize at various agricultural meteorological stations merely provides an approximation of the actual harvest date. Enhancing the precision of precipitation statistics could yield more objective evaluation results. In addition, the research scale of this study was refined to include county-level units in the north‒south transitional zone of China. Due to difficulties in obtaining complete and long-term county-level yield data for winter wheat and summer maize, we did not conduct a correlation analysis between the climate data and yield data but analysed only the stress of extreme precipitation and drought based on the natural occurrence of disasters. In our subsequent research, we will comprehensively consider both natural and socioeconomic factors to develop a risk assessment system that incorporates the combined impact of disaster stress, exposure, and disaster resilience. By collecting comprehensive data at the county level, our aim is to evaluate climate-related disaster risks in this region more accurately and provide systematic guidance for targeted prevention and mitigation of agricultural production risks in different areas.

## Data Availability

The data that support the findings of this study are available upon reasonable request from the corresponding author.
